# Comparative Studies of Antimicrobial Resistance in *Escherichia coli*, *Salmonella*, and *Campylobacter* Isolates from Broiler Chickens with and without Use of Enrofloxacin

**DOI:** 10.3390/foods12112239

**Published:** 2023-06-01

**Authors:** Ke Shang, Ji-Hyuk Kim, Jong-Yeol Park, Yu-Ri Choi, Sang-Won Kim, Se-Yeoun Cha, Hyung-Kwan Jang, Bai Wei, Min Kang

**Affiliations:** 1 College of Animal Science and Technology, Luoyang Key Laboratory of Live Carrier Biomaterial and Animal Disease Prevention and Control, Henan University of Science and Technology, Luoyang 471000, China; shangke0624@gmail.com; 2 Department of Animal Resources Science, Kongju National University, Yesan 32439, Republic of Korea; jihyuk@kongju.ac.kr; 3 Department of Avian Diseases, College of Veterinary Medicine and Center for Avian Disease, Jeonbuk National University, Iksan 54596, Republic of Korea; jyp410@naver.com (J.-Y.P.); 9cinderella7@naver.com (Y.-R.C.); sk970221@gmail.com (S.-W.K.); seyeouncha@gmail.com (S.-Y.C.); hkjang@jbnu.ac.kr (H.-K.J.); 4 Bio Disease Control (BIOD) Co., Ltd., Iksan 54596, Republic of Korea

**Keywords:** *E. coli*, *Salmonella*, *Campylobacter*, enrofloxacin, antimicrobial resistance, broiler chicken, field trial

## Abstract

This study investigated the effect of enrofloxacin (ENR) administration on the prevalence and antimicrobial resistance of *E. coli*, *Salmonella*, and *Campylobacter* isolated from broiler chickens under field conditions. The isolation rate of *Salmonella* was significantly lower (*p* < 0.05) on farms that administered ENR (6.4%) than on farms that did not (11.6%). The *Campylobacter* isolation rate was significantly higher (*p* < 0.05) in farms that administered ENR (6.7%) than in farms that did not (3.3%). The ratio of resistance to ENR was significantly higher (*p* < 0.05) in *E. coli* isolates from farms that used ENR (88.1%) than farms that did not (78.0%). The respective ratio of resistance to ampicillin (40.5% vs. 17.9%), chloramphenicol (38.0% vs. 12.5%), tetracycline (63.3% vs. 23.2%), and trimethoprim/sulfamethoxazole (48.1% vs. 28.6%) and the ratio of intermediate resistance to ENR (67.1% vs. 48.2%) were significantly higher (*p* < 0.05) in *Salmonella* isolates from the farms that used ENR than farms that did not. In conclusion, the use of ENR at broiler farms was an important factor in decreasing the prevalence of *Salmonella* but not *Campylobacter* and caused ENR resistance among *E. coli* and *Salmonella* but not *Campylobacter*. Exposure to ENR could have a co-selective effect on antimicrobial resistance in enteric bacteria in the field.

## 1. Introduction

In many countries, antimicrobial resistance in foodborne pathogens is recognized as an accumulative problem [1,2]. Systemically administered antimicrobial agents may cause the emergence and spread of antimicrobial resistance among zoonotic foodborne pathogens, such as non-typhoid *Salmonella* serotypes and *Campylobacter* [3,4]. *Salmonella enterica* and *Campylobacter* infections are the major causes of foodborne gastroenteritis in humans (known as salmonellosis and campylobacteriosis, respectively) in many countries [4,5,6,7]. *Salmonella* causes gastroenteritis with clinical symptoms such as headache, abdominal pain, and diarrhea in both adults and children; campylobacteriosis frequently occurs in children younger than five years of age, and *Campylobacter* species cause a wide range of syndromes, from asymptomatic infections to severe systemic infections. In addition, the major source of *Salmonella* and *Campylobacter* infections in humans has been identified as poultry, as a reservoir of foodborne enteric pathogens and antimicrobial-resistant bacteria [8,9]. Fluoroquinolones (FQs) are the primary treatment against these bacteria [10].

Fluoroquinolones belong to a family of synthetic antimicrobials with a broad spectrum of activity. Nalidixic acid, which does not contain fluorine, is the parent compound of the quinolone family, which includes FQs. The FQs are one of the few therapeutics available for the treatment of campylobacteriosis, which is transmitted to humans through animal sources, such as poultry [11,12]. Given this importance, FQs are classified by the World Health Organization (WHO) as critically important antimicrobials (for human medicine) and veterinary critically important antimicrobial agents (for animals) that directly inhibit DNA replication and transcription [13,14]. Several mechanisms facilitate bacterial FQ resistance, including mutation(s) at the drug binding sites in the quinolone resistance determining region (QRDR) of *gyr*A, *gyr*B, *par*A, and *par*C [15], reduced accumulation of FQs in cells [16,17], and plasmid mediated quinolone resistance (PMQR) genes [*qnr* family, *aac*(6′)-Ib-cr, *oqx*AB, and *qep*A genes] [18]. Such resistance is usually associated with a single point mutation in the *gyr*A gene [19].

Fluoroquinolones are used in the treatment of animal and human diseases [20]. In veterinary medicine, especially in broiler fattening, they are ordinarily used to treat colibacillosis, with oral administration being the preferred route [21]. Despite their efficacy, the use of FQs in veterinary medicine is controversial. As such, the use of FQs intensifies FQ resistance in foodborne pathogens and thus poses a public health problem [22]. The escalating FQ resistance in foodborne pathogens may be caused by using antimicrobials in veterinary medicine, as food animals are the main reservoir for these pathogens [23]. FQ resistance (via oral administration) has been found in *Salmonella*, *Campylobacter*, and *Escherichia coli* (*E. coli*) in broilers [24,25]. Antimicrobial resistance is a major problem for the intestinal health of humans and other animals. In 2017, the WHO listed FQ-resistant *Salmonella* and *Campylobacter jejuni* among the top nine bacterial pathogens, for which new antimicrobials are urgently needed [26]. The use of FQs to treat diseases in food animals has led to the selection of FQ-resistant strains of *Campylobacter*, especially in poultry [15]. FQ resistance develops rapidly among bacteria in poultry treated with FQ [27,28,29,30]. Contamination of food with *Salmonella* and *Campylobacter* remains a significant food safety concern [31], and antimicrobial-resistant (AMR) *Salmonella* and *Campylobacter* strains pose an additional threat to food safety. FQ-resistant *Campylobacter* is found in poultry in most regions of the world [8]. In addition, the increasing use of antimicrobials, especially FQs, likely increases the incidence of foodborne infections with AMR *Salmonella* [1].

Enrofloxacin (ENR), a second-generation FQs, is commonly used in chickens because of its favorable pharmacokinetic profile and its excellent activity against gram-negative aerobic bacteria and some gram-positive bacteria. Prophylactic treatment of poultry with ENR, an FQ antibacterial used to treat infections in animals, has been associated with increasing resistance to ciprofloxacin, posing a risk to human health [28,32,33]. Despite its efficacy, some countries, such as the USA, Finland, and Denmark, have banned the use of FQ in poultry [34,35]. In Poland and most countries of the European Union, therapeutic use of FQ in poultry is still allowed [36]. Concerns about the entry of zoonotic AMR bacteria into the food chain and resulting human infections led the Food and Drug Administration (FDA) to ban the use of ENR in poultry in the USA in September 2005. The EU has established maximum residue limits (Regulation CE N. 2377/90) [37]. In Korea, ENR has been progressively utilized to treat bacterial infections in chickens since it was approved for veterinary use in 1987 [38]. In fact, ENR was the best-selling antimicrobial agent for chickens in Korea. In addition, the use of all antibiotic feed additives classified as growth promoters has been banned in Korea since July 2011, and the use of ENR in commercial laying hens has been prohibited since May 2017 [39,40].

The bacterial species selected for the present study (*Campylobacter*, *Salmonella*, and *E. coli*) are targets of FQs in human medicine or veterinary medicine, and data on FQ resistance in these bacterial species span several years [41]. Treatment of livestock with ENR leads to a rapid selection of *Campylobacter*, *E. coli* [42,43,44], and *Salmonella* [36] which are less susceptible to these antibiotics. Nevertheless, few studies have focused on the relationship between ENR use and ENR resistance in foodborne pathogens from broilers in the field [45]. In this field trial, we investigated the relationship between ENR use and the emergence of ENR resistance in *Salmonella* and *Campylobacter*, whereas *E. coli* was used as an indicator bacterium.

## 2. Materials and Methods

### 2.1. Studied Isolates

There were two groups of farms (Group 1—contained farms that use ENR; and Group 2—contained farms that do not use ENR). *E. coli* isolates (*n* = 516; 306 and 210 isolates from Group 1 and Group 2, respectively), *Salmonella* isolates (*n* = 255; 134 and 121 isolates from Group 1 and Group 2, respectively), and *Campylobacter* isolates (*n* = 165; 132 and 33 isolates from Group 1 and Group 2, respectively) were sequestered and assessed. All isolates were from 2418 clinical samples composed of fecal (1-day-old and 15–25-days-old broiler chickens) or from environmental source (litter, feed, and water) samples collected from broiler farms or meat samples collected from downstream retail markets (Table S1). All samples were collected between September and December in 2015. In Group 1, 0.5 g/L ENR was constantly added to the drinking water of 2–4-day-old chicks. *Salmonella*, *E. coli*, and *Campylobacter* were isolated using the standard method described previously [45,46,47,48].

No endangered population was involved, and no endangered species were used for the experiments. No chickens were touched or killed; fecal samples were collected by a veterinarian with prior approval from the farm managers. Therefore, no ethical approval was required for this study.

### 2.2. Antimicrobial Susceptibility Testing

For *E. coli* and *Salmonella* isolates, the minimum inhibitory concentrations (MICs) of 14 antimicrobial agents, including nalidixic acid (2–128 μg/mL), ciprofloxacin (0.12–16 μg/mL), neomycin (2–32 μg/mL), gentamicin (1–64 μg/mL), streptomycin (2–128 μg/mL), tetracycline (2–128 μg/mL), amoxicillin/clavulanic acid (2/1–64/32 μg/mL), cefoxitin (1–32 μg/mL), ceftiofur (0.5–8 μg/mL), ampicillin (2–32 μg/mL), trimethoprim/sulfamethoxazole (0.12/0.38–4/76 μg/mL), colistin (2–32 μg/mL), florfenicol (2–64 μg/mL), and chloramphenicol (2–64 μg/mL) were determined using the KRNV4F Sensititre Panel (TREK Diagnostic Systems, Incheon, Korea), and the MIC of ENR (0.125–128 μg/mL) was verified by the agar dilution method (Table S2) [49]. KRNV4F Sensititre panel made by Trek Diagnostic Systems is ready-to-use 96-well microplate coated with 15 kinds of antimicrobial substances with multiple dilution concentration using Mueller–Hinton broth (MHB) medium. *E. coli* (ATCC 25922) was used as a quality control strain. The susceptibility breakpoints of most antimicrobials were interpreted based on Clinical and Laboratory Standards Institute (CLSI) guidelines [50] or other references [49,51,52].

The susceptibility of all *Campylobacter* isolates to 11 antimicrobial agents was ascertained using the Campy Sensititre Panel (TREK) and the agar dilution method. Campy Sensititre Panel made by Trek Diagnostic Systems is ready-to-use 96-well microplate coated with nine kinds of antimicrobial substances detecting for *Campylobacter*. Cation-adjusted Mueller–Hinton broth with TES buffer with 5% lysed horse blood medium was used in this method. Susceptibility to two antimicrobial agents (not available in this plate), namely ENR (0.125–128 μg/mL) and ampicillin (8–128 μg/mL), were confirmed by the standard agar dilution method (Table S2) [49]. The remaining nine antimicrobials were tested with Sensititre plates containing azithromycin (0.015–64 μg/mL), erythromycin (0.03–64 μg/mL), telithromycin (0.015–8 μg/mL), nalidixic acid (4–64 μg/mL), ciprofloxacin (0.015–64 μg/mL), clindamycin (0.03–16 μg/mL), gentamicin (0.12–32 μg/mL), florfenicol (0.03–64 μg/mL), and tetracycline (0.06–64 μg/mL). Breakpoints were established according to the National Antimicrobial Resistance Monitoring System criteria [51]. Because there were no ampicillin and ENR breakpoints for *Campylobacter*, we used the breakpoints for *Enterobacteriaceae* from the CLSI criteria [49,50]. *Campylobacter jejuni* ATCC 33560 was used as the reference quality control. Isolates that were resistant to at least three antimicrobial classes were classified as multidrug-resistant (MDR).

The MIC_50_ and MIC_90_ values, as well as the range of values obtained, are important parameters for reporting the results of susceptibility testing when multiple isolates of a given species are investigated. The MIC_50_ represents the MIC value at which ≥50% of the isolates in a test population are inhibited; it corresponds to the mean MIC value. The MIC_90_ represents the MIC value at which ≥90% of the strains within a test population are inhibited [53].

### 2.3. Molecular Characterization of Antimicrobial Resistance

For molecular characterization of quinolone resistance genes, the quinolone-resistant isolates were screened for plasmid-mediated quinolone resistance (PMQR) genes by PCR and sequencing. These genes included *qnr*A, *qnr*B, *qnr*S, *qnr*D, *qep*A, *oqx*A, *aac*(6′)-lb-cr, and mutations in the quinolone resistance determining region (QRDR), and *gyr*A, *gyr*B, *par*C, and *par*E as previously described [54,55]. Positive controls were used in all PCR reactions (Table S3). PCR products were purified using a QIAquick Gel Extraction Kit (Qiagen, Hilden, Germany) and then directly sequenced (SolGent Co., Ltd., Daejeon, Republic of Korea) for protein sequence analysis and alignment using the program BLAST (www.ncbi.nlm.nih.gov/BLAST/) accessed on 1 March 2022.

### 2.4. Statistical Analysis

Statistical analysis was performed using the SPSS program version 19.0 for Windows. The Chi-square test was used to analyze significant differences in isolation rates of *Salmonella* and *Campylobacter* and AMR rates of *E*. *coli*, *Salmonella*, and *Campylobacter* between broilers from Groups 1 and 2. *p*-values < 0.05 were considered indicative of statistical significance. Results are reported as the mean with 95% confidence interval (CI).

## 3. Results

### 3.1. Prevalence of Salmonella and Campylobacter in the Presence or Absence of ENR Use

*Salmonella* isolation rates from 15–25-days-old broilers and from retail meat were significantly lower (*p* < 0.001) in Group 1 than in Group 2 (Table 1). There was no significant difference in the *Salmonella* isolation rate of 1-day-old broilers between Group 1 and Group 2. *Campylobacter* isolation rates of 15–25-days-old broilers and retail meat were significantly higher (*p* < 0.05) in Group 1 than in Group 2. The *Campylobacter* isolation rate of 1-day-old broilers was similar between Group 1 and Group 2.

### 3.2. Antimicrobial Resistance of E. coli, Salmonella, and Campylobacter Isolates in the Presence or Absence of ENR Use

The prevalence of antimicrobial resistance differed between Group 1 and Group 2 for 16 antimicrobials assessed (Table 2, Table 3 and Table 4). *E. coli* isolates from 15–25-days-old broilers from Group 1 were significantly more likely to be resistant to cephalothin (*p* = 0.038) and ENR (*p* = 0.048) (Table 2). The prevalence of multidrug resistance in *E. coli* isolates did not differ significantly between Group 1 and Group 2. A higher frequency of *Salmonella* isolates from 15–25-days-old broilers from Group 1 were resistant to ampicillin (*p* = 0.005), chloramphenicol (*p* = 0.001), tetracycline (*p* < 0.001), and intermediate resistance (IR) to ENR (*p* = 0.028) (Table 3). The prevalence of multidrug resistance in *Salmonella* isolates was also significantly higher in 15–25-days-old broilers in Group 1 (*p* = 0.016). *Campylobacter* isolates from 15–25-days-old broilers from Group 1 had significantly lower resistance to tetracycline (*p* < 0.001). The prevalence of multidrug resistance in *Campylobacter* isolates was also significantly lower on Group 1 (*p* = 0.005) (Table 4).

### 3.3. Distribution of the MIC_50_/MIC_90_ Values of (Fluoro)Quinolones among E. coli, Salmonella, and Campylobacter Isolates from Untreated or ENR-Treated Broilers

The MIC_50_/MIC_90_ values of *E. coli*, *Salmonella*, and *Campylobacter* isolates from the broilers with or without ENR treatment are illustrated in Tables S3–S5. The MIC_50_ of ciprofloxacin and ENR were 8 μg/mL and 16 μg/mL against *E. coli* isolates from both 1-day-old broilers and retail meat in Group 1, respectively, which was higher than the 4 μg/mL and 8 μg/mL, respectively, in Group 2 (Table S4). Similarly, the MIC_50_ of ENR against the *Salmonella* isolates from ENR-treated and untreated 15–25-days-old broilers were 0.5 and <0.25 μg/mL, respectively. The MIC_90_ of ciprofloxacin against *Salmonella* isolates from ENR-treated and untreated 15–25-days-old broilers was 0.5 and 0.25 μg/mL, respectively (Table S5). There was no enhanced MIC_50_/MIC_90_ value against *Campylobacter* isolates from broilers in Group 1 as compared with isolates from broilers in Group 2 (Table S6). On the contrary, the MIC_50_ of ciprofloxacin and ENR were 16 μg/mL and 8 μg/mL against *E. coli* isolates from retail meat of broilers in Group 2, which was higher than that of 8 μg/mL and 4 μg/mL against the isolates from in Group 1, respectively. The MIC_90_ of ENR were 32 μg/mL and 16 μg/mL against *Campylobacter* isolates from 15–25-days-old broilers and retail meat of broilers in Group 2 and 16 μg/mL and 8 μg/mL against broilers in Group 1, respectively.

### 3.4. Prevalence of PMQR and QRDR Mutations among the Selected E. coli, Salmonella, and Campylobacter Isolates from Untreated or ENR-Treated Broilers

The prevalence of PMQR and QRDR mutations in *E. coli* isolates (ENR, MIC ≥ 1) is displayed in Table 5. PMQR genes were not detected in any isolate; *qnr*B and *qnr*S1 genes were revealed in *E. coli* isolates from broilers with ENR treatment. The most frequent QRDR pattern in *E. coli* isolates (ENR MIC, 16–32 µg/mL) from broilers with ENR treatment was Ser-83-Leu and Asp-87-Asn in *gyr*A and Ser-80-Ile and Ser-83-Tyr in *par*C (8/28, 28.6%). The most common QRDR patterns observed in *E. coli* isolates (ENR MIC, 8–16 µg/mL) from broilers without ENR treatment were Ser-80-Ile and Ser-83-Tyr in *par*C (6/17, 35.3%). Alterations in the *gyr*A gene were frequently observed in 92.9% (26/28) and 76.5% (13/17) of *E. coli* isolates from broilers in Group 1 and Group 2, respectively. Alterations in the *par*C gene were noted in all *E. coli* isolates. Substitutions in the *par*E gene occurred less often, with 85.7% (24/28) and 82.4% (14/17) of isolates from broilers in Group 1 and Group 2, respectively, having wild type *par*C.

The *gyrA* gene sequences of targeted isolates were submitted to the GenBank database and are available under the accession numbers OR047806-OR047961; the *parC* gene sequences of targeted isolates were submitted to the GenBank database and are available under the accession numbers OR047672-OR047805.

The prevalence of PMQR and QRDR mutations in *Salmonella* isolates (ENR, MIC ≥ 0.25) is indicated in Table 6. PMQR genes were not observed in any *Salmonella* isolate. The most common QRDR pattern detected in *Salmonella* isolates (ENR MIC, 0.25–1.00 µg/mL) from broilers in Group 1 was Asp-87-Gly in *gyr*A and Tyr-57-Ser in *par*C (28/50, 56.0%); the same QRDR pattern was observed in *Salmonella* isolates (ENR MIC, 0.25–0.50 µg/mL) from broilers in Group 2 (33/49, 67.3%). Changes in the *gyr*A gene were observed in 90.0% (45/50) and 98.0% (48/49) of *Salmonella* isolates from broilers in Group 1 and Group 2, respectively. In *Salmonella* isolates from broilers in Group 1 and Group 2, 92.0% (46/50) and 98.0% (48/49) of the changes in the *par*C gene were observed, respectively.

The prevalence of PMQR and QRDR mutations among *Campylobacter* isolates (ENR, MIC ≥ 4) is presented in Table 7. PMQR genes were not detected in any *Campylobacter* isolate. The only QRDR pattern of Thr-86-Ile in *gyr*A was observed in all *Campylobacter* isolates (ENR MIC, 4–32 µg/mL) from broilers in Group 1 and Group 2.

## 4. Discussion

The development of antimicrobial resistance indicates a clear link between the use of antimicrobials in veterinary medicine and animal production [56]. Administration of FQs such as ENR to poultry can lead to rapid selection of *E. coli*, *Salmonella*, and *Campylobacter*, which are less susceptible to such antimicrobials. In the present study, the interaction between the use of ENR and antimicrobial resistance in *E. coli*, *Salmonella*, and *Campylobacter* of broiler chickens was investigated under field conditions.

As expected, the isolation rate of *Salmonella* from samples of 15–25-days-old broilers was significantly lower (*p* < 0.05) in Group 1 than in Group 2 (56/485, 11.6%) (Table 1). The isolation rate of *Salmonella* from broiler meat was also significantly lower (*p* < 0.05) in Group 1 (30/105, 28.6%) than in Group 2 (39/64, 60.9%). Therefore, the use of ENR was an important factor leading to a lower prevalence of *Salmonella* in both broiler farms and the retail market, which is consistent with a previous study [46]. In contrast, *Campylobacter* isolation rates from samples of 15–25-days-old broilers were significantly higher (*p* < 0.05) in Group 1 (83/1236, 6.7%) than in Group 2 (16/485, 3.3%). Similarly, *Campylobacter* isolation rates from broiler meat were significantly higher (*p* < 0.05) in Group 1 (48/105, 45.7%) than in Group 2 (17/64, 26.6%). Instead, the use of ENR increased *Campylobacter* prevalence in both broiler farms and the retail market. That could be because *Campylobacter* resistance to FQs has already reached a high level, and the use of ENR did not reduce prevalence as it did for *Salmonella* [55]. However, a previous study demonstrated a low prevalence of *Campylobacter* in retail chickens after the ENR ban [37]. In the present study, the use of ENR at the broiler stage had a different effect on the prevalence of *Salmonella* and *Campylobacter* in broilers and their meat products such that ENR use resulted in a high positive rate of *Campylobacter* but a low positive rate of *Salmonella*. The reasons for the above phenomena might be that the ENR resistance of *Salmonella* and *Campylobacter* isolates was different [47,55]. Moreover, low and high ENR resistance values were observed in *Salmonella* (Table 3) and *Campylobacter* isolates (Table 4), respectively. Therefore, the use of FQs in chickens rapidly leads to the selection of resistant *Campylobacter* organisms. However, FQ-resistant *Campylobacter* isolates are counter-selected under field conditions in the absence of selection pressure [25,29].

After administration of ENR, a significant change in resistance to ENR was observed in *E. coli* isolates from 15–25-days-old broilers. In the present study, we discovered a significant difference (*p* = 0.048) in resistance to ENR in *E. coli* isolates from 15–25-days-old broilers between Group 1 (89/101, 88.1%) and Group 2 (92/118, 78.0%) farms (Table 2). Therefore, ENR use may be an important factor in ENR resistance of *E. coli* in broiler farms. Similarly, antimicrobial resistance in *E. coli* is mainly drug-dependent [45]. Resistance to cefoxitin and tetracycline was significantly higher in *E. coli* isolates from broiler meat in Group 2 (45.0%; 100%) than in broilers in Group 1 (20.5%; 64.1%). This observation could be due to cross-contamination by *E. coli* from resistant carrier chickens slaughtered on the same day or contamination by resident flora with AMR in the slaughterhouse [47]. These data are consistent with previously published studies indicating that antimicrobial resistance in broilers not exposed to ENR showed changes over time [44,57].

There was no significant difference (*p* > 0.05) in resistance to FQs in *Salmonella* isolates from 15–25-days-old broilers between Group 1 (ciprofloxacin, 1.3%; ENR, 2.5%) and Group 2 (0%) farms (Table 3). This result could be due to low FQ resistance of *Salmonella* isolates from both Group 1 and Group 2, likely because of the short growth cycle of modern broilers, which does not allow sufficient time for resistance to develop, even after antimicrobial treatment. *Salmonella* isolates from 15–25-days-old broilers in Group 1 revealed a significant increase (*p* = 0.028) in intermediate resistance to ENR after ENR administration. A significant escalation (*p* < 0.05) in resistance to ampicillin, chloramphenicol, tetracycline, and trimethoprim/sulfamethoxazole was also observed in *Salmonella* isolates from 15–25-days-old broilers in Group 1. Moreover, the results of *E. coli* resistance to ampicillin, chloramphenicol, tetracycline, and trimethoprim/sulfamethoxazole were also consistent. The plausible explanations of the above phenomena were that the four antimicrobials were commonly used in broiler farms, and resistance to these antimicrobials was most frequently observed in isolates from chickens. In fact, selection pressure exerted by the antimicrobial agent contributed to the co-selection of this antimicrobial resistance pattern; the co-selection of resistance to more than one antimicrobial agent is a common feature of resistance acquired by horizontal gene transfer due to the genetic linkage of resistance genes [45,58,59].

There was no significant difference (*p* > 0.05) in resistance to FQs in *Campylobacter* isolates from broilers between Group 1 (78.4%) and Group 2 (100%) farms (Table 4). This may be due to the high FQ resistance of *Campylobacter* isolated from both the ENR-treated and untreated broilers, making it difficult to discern differences in FQ resistance between the two populations. A previous study demonstrated a low rate of ciprofloxacin resistance in *Campylobacter* in retail chickens after the ENR ban [36]. In other previous studies, therapeutic administration of ENR to mice did not result in FQ resistance in *Campylobacter jejuni* [60]. However, treatment of pigs with FQs selected and spread FQ-resistant *Campylobacter* in the house [61].

In general, PMQR genes confer low resistance to quinolones but may play an important role in the emergence of resistance mutations and the spread of antimicrobial resistance [62]. In the present study, the PMQR genes (*qnr*B and *qnr*S1) were detected in *E. coli* isolates from the broilers in Group 1, whereas these genes were absent in *E. coli* isolates from the broilers in Group 2 (Table 5). Accordingly, the *qnr*S1 gene was uncovered in *E. coli* isolates from chicken meat produced in integrated broiler operations in Korea [63]. In the present study, more amino-acid mutations in the *gyr*A, *par*C, and *par*E genes were discovered in broiler isolates from Group 1 than in isolates from Group 2. Thus, the use of ENR resulted in the emergence of PMQR genes and higher resistance to ENR with more mutations in QRDR genes.

Although PMQR has been extensively studied, QRDR mutations seem to represent the main mechanism of quinolone resistance in animal isolates [64]. Moreover, PMQR was commonly detected in Enterobacteriaceae, particularly in *E. coli*, and the prevalence of PMQRs in *Salmonella* remains extremely low [65]. In the present study, no PMQR gene was realized in all *Salmonella* (*n* = 99) and *Campylobacter* (*n* = 76) isolates tested (Table 6 and Table 7). The most common QRDR pattern, an exchange of a single amino acid in both *gyr*A (Asp-87-Gly) and *par*C (Tyr-57-Ser), was identical in *Salmonella* isolates from broilers in Group 1 and Group 2 (Table 6), and similar QRDR patterns were noted, except for the Ser-83-Ala exchange in the *gyr*A gene in *Salmonella* isolates from Group 1. Only one QRDR pattern (Thr-86-Ile in *gyr*A) was revealed in the *Campylobacter* isolates from both Group 1 and Group 2 (Table 7). This is consistent with our previous findings that the Thr-86-Ile substitution in the *gyrA* was the primary contributor to the high-level quinolone resistance in *Campylobacter* isolates from duck meats [66]. Therefore, the use of ENR may not lead to the emergence of PMQR genes and QRDR genes with amino acid exchange in *Salmonella* and *Campylobacter* isolates [67].

## 5. Conclusions

In conclusion, ENR use in broiler farms is an important factor in reducing the prevalence of *Salmonella* but not *Campylobacter*, as well as causing ENR resistance in *E. coli* but not in *Salmonella* or *Campylobacter*. ENR use in broiler farms may lead to the emergence of PMQR genes and high levels of QRDR mutations and ENR resistance in *E. coli* and less so in *Salmonella* and *Campylobacter*. Understanding the relationship between antimicrobial use in food-producing animals and the emergence of resistant commensal bacteria may facilitate the prudent use of antimicrobials in these animals. Therefore, because of its selective effect, ENR must be used judiciously.

## Figures and Tables

**Table 1:** Prevalence of *Salmonella* and *Campylobacter* in untreated or ENR-treated broiler chickens.

Pathogens	Group	1-Day-Old			15–25-Days-Old			Retail Meat		
		No. of Samples	No. of Isolates	Isolation Rate (%)	No. of Samples	No. of Isolates	Isolation Rate (%)	No. of Samples	No. of Isolates	Isolation Rate (%)
*Salmonella*	1	308	25	8.1 (5.1–11.2)	1236	79	6.4 (5.0–7.8)	105	30	28.6 (19.9–37.2)
	2	220	26	11.8 (7.6–16.1)	485	56	11.6 (8.7–14.4)	64	39	60.9 (49.0–72.9)
	*p*-value			0.114			**<0.001**			**<0.001**
*Campylobacter*	1	308	1	0.3 (0.0–1.0)	1236	83	6.7 (5.2–8.1)	105	48	45.7 (36.2–55.2)
	2	220	0	0.0 (0.0–0.0)	485	16	3.3 (1.7–4.9)	64	17	26.6 (15.7–37.4)
	*p*-value			0.720			**0.006**			**0.013**

Bold type indicates *p*-value < 0.05; ENR: enrofloxacin; results are expressed as the mean (95% confidence interval). Group 1—contained farms that use ENR; and Group 2—contained farms that do not use ENR. In Group 1, 0.5 g/L ENR was constantly added to the drinking water of 2–4-day-old chicks.

**Table 2:** Antimicrobial resistance of *E. coli* isolates from untreated or ENR-treated broiler chickens.

Antimicrobial Class	Antimicrobials	Antimicrobial Resistance (%)								
		1-Day-Old			15–25-Days-Old			Retail Meat		
		Group 1	Group 2	*p*-Value	Group 1	Group 2	*p*-Value	Group 1	Group 2	*p*-Value
Combinations	Amoxicillin/clavulanic acid	9.1	9.1	1.000	6.1	5.4	0.838	23.1	45.0	0.083
Penicillin	Ampicillin	63.6	72.9	0.316	88.6	82.2	0.181	94.9	70.0	**0.025**
Cephalosporins	Cephalothin	100.0	40.0	**<0.001**	54.7	39.6	**0.038**	37.9	60.0	0.128
	Cefoxitin	9.1	8.0	1.000	9.4	10.9	0.734	20.5	45.0	**0.049**
	Ceftiofur	11.4	11.9	1.000	20.0	16.9	0.557	46.2	60.0	0.314
Amphenicols	Chloramphenicol	25.0	25.4	1.000	61.0	52.5	0.206	59.0	55.0	0.770
	Florfenicol	18.2	23.7	0.662	55.2	50.4	0.487	80.6	47.1	**0.016**
Aminoglycosides	Streptomycin	43.2	54.2	0.267	67.6	66.9	0.915	43.6	55.0	0.406
	Gentamicin	9.1	8.5	1.000	26.0	17.8	0.140	61.5	25.0	**0.017**
	Neomycin	2.3	8.5	0.366	14.6	8.5	0.166	0.0	0.0	N/A
Tetracycline	Tetracycline	75.0	62.7	0.186	76.0	82.2	0.252	64.1	100.0	**0.006**
Sulfonamides	Trimethoprim/sulfamethoxazole	29.5	34.0	0.692	40.6	40.0	0.931	76.9	80.0	1.000
Quinolones	Nalidixic acid	100.0	81.4	**0.007**	99.0	96.6	0.439	82.1	85.0	1.000
	Ciprofloxacin	72.7	55.9	0.081	81.9	73.7	0.144	82.1	70.0	0.290
	Enrofloxacin	68.2	59.3	0.357	88.1	78.0	**0.048**	79.5	80.0	1.000
Polymyxin	Colistin	0.0	0.0	N/A	1.0	0.8	1.000	2.6	0.0	1.000
MDR		77.3	66.1	0.612	95.2	89.8	0.763	100.0	100.0	1.000

N/A, not available; bold type indicates *p*-value < 0.05. Group 1—contained farms that use ENR; and Group 2—contained farms that do not use ENR. In Group 1, 0.5 g/L ENR was constantly added to the drinking water of 2–4-day-old chicks.

**Table 3:** Antimicrobial resistance of *Salmonella* isolates from untreated or ENR-treated broiler chickens.

Antimicrobial Class	Antimicrobials	Antimicrobial Resistance (%)								
		1-Day-Old			15–25-Days-Old			Retail Meat		
		Group 1	Group 2	*p*-Value	Group 1	Group 2	*p*-Value	Group 1	Group 2	*p*-Value
Combinations	Amoxicillin/clavulanic acid	0.0	0.0	N/A	1.3	0.0	1.000	0.0	0.0	NA
Penicillin	Ampicillin	8.0	0.0	0.453	40.5	17.9	**0.005**	56.7	35.9	0.140
Cephalosporins	Cephalothin	0.0	0.0	N/A	2.5	1.8	1.000	6.7	0.0	0.361
	Cefoxitin	20.0	11.5	0.656	7.6	7.1	1.000	0.0	2.6	1.000
	Ceftiofur	8.0	3.8	0.972	6.3	3.6	0.750	0.0	7.7	0.338
Amphenicols	Chloramphenicol	0.0	0.0	N/A	38.0	12.5	**0.001**	56.7	35.9	0.140
	Florfenicol	0.0	0.0	N/A	22.8	12.5	0.130	20.0	23.1	0.990
Aminoglycosides	Streptomycin	36.0	26.9	0.692	49.4	44.6	0.588	26.7	28.2	1.000
	Gentamicin	0.0	0.0	N/A	1.3	1.8	1.000	0.0	0.0	0.055
	Neomycin	60.0	34.6	0.125	41.8	44.6	0.740	16.7	41.0	0.155
Tetracycline	Tetracycline	24.0	11.5	0.424	63.3	23.2	**<0.001**	53.3	33.3	0.404
Sulfonamides	Trimethoprim/sulfamethoxazole	0.0	3.8	1.000	48.1	28.6	**0.022**	56.7	43.6	1.000
Quinolones	Nalidixic acid	100.0	96.2	1.000	89.9	100.0	**0.037**	96.7	97.4	1.000
	Ciprofloxacin-R	0.0	0.0	N/A	1.3	0.0	1.000	3.3	0.0	1.000
	Ciprofloxacin-IR	48.0	19.2	0.060	58.2	55.4	0.740	66.7	87.2	0.080
	Enrofloxacin-R	0.0	0.0	N/A	2.5	0.0	0.634	0.0	5.1	1.000
	Enrofloxacin-IR	48.0	26.9	0.120	67.1	48.2	**0.028**	73.3	69.2	0.710
Polymyxin	Colistin	0.0	0.0	N/A	6.3	1.8	0.402	0.0	2.6	1.000
MDR		28.0	23.1	0.765	69.6	32.1	**0.016**	56.7	56.4	0.991

N/A, not available; bold type indicates *p*-value < 0.05; R, resistance; IR, intermediate resistance. Group 1—contained farms that use ENR; and Group 2—contained farms that do not use ENR. In Group 1, 0.5 g/L ENR was constantly added to the drinking water of 2–4-day-old chicks.

**Table 4:** Antimicrobial resistance of *Campylobacter* isolates from untreated or ENR-treated broiler chickens.

Antimicrobial Class	Antimicrobials	Antimicrobial Resistance (%)								
		1-Day-Old			15–25-Days-Old			Retail Meat		
		Group 1	Group 2	*p*-Value	Group 1	Group 2	*p*-Value	Group 1	Group 2	*p*-Value
Penicillin	Ampicillin	100.0	-	N/A	66.7	56.3	0.448	39.6	82.4	**0.002**
Tetracycline	Tetracycline	0.0	-	N/A	15.7	81.3	**<0.001**	58.3	76.5	0.183
Macrolides	Azithromycin	0.0	-	N/A	0.0	0.0	N/A	2.1	0.0	0.549
	Erythromycin	0.0	-	N/A	0.0	0.0	N/A	0.0	0.0	N/A
Amphenicols	Florfenicol	0.0	-	N/A	0.0	0.0	N/A	2.1	0.0	N/A
Aminoglycosides	Gentamicin	0.0	-	N/A	0.0	0.0	N/A	0.0	0.0	N/A
Ketolides	Telithromycin	0.0	-	N/A	0.0	0.0	N/A	0.0	0.0	N/A
Lincosamides	Clindamycin	0.0	-	N/A	0.0	0.0	N/A	0.0	0.0	N/A
Quinolones	Nalidixic acid	0.0	-	N/A	78.4	100.0	0.054	95.8	88.2	0.263
	Ciprofloxacin	0.0	-	N/A	78.4	100.0	0.054	100.0	100.0	N/A
	Enrofloxacin	0.0	-	N/A	78.4	100.0	0.054	100.0	100.0	N/A
MDR		0.0	-	N/A	11.8	43.8	**0.005**	20.8	58.8	**0.004**

N/A, not available; -, indicates no isolate; bold type indicates *p*-value < 0.05. Group 1—contained farms that use ENR; and Group 2—contained farms that do not use ENR. In Group 1, 0.5 g/L ENR was constantly added to the drinking water of 2–4-day-old chicks.

**Table 5:** Prevalence of PMQR and amino acid changes in the *gyr*A, *par*C, and *par*E genes of *E. coli* isolates (*n* = 45) from Group 1 and Group 2 or the corresponding MICs of ENR ^a^.

Group ^b^ (*n*)	*n* (%) ^c^	ENR MIC (µg/mL)	PMQR (%)	QRDR Pattern												
				*gyr*A						*par*C					*par*E	
Group 1 (*n* = 28)	8 (28.6)	16–32	0.0	Ser-83-Leu	Asp-87-Asn	^e^				Ser-80-Ile		Ser-83-Tyr			*wt*	
	7 (25.0)	4–16	*qnrB* (14.3)	*wt* ^d^						Ser-80-Ile		Ser-83-Tyr			*wt*	
	2 (7.1)	4	0.0	Ser-83-Leu								Ser-83-Tyr			*wt*	
	2 (7.1)	32	0.0	Ser-83-Leu	Asp-87-Asn					Ser-80-Ile		Ser-83-Tyr			Ser-458-Ala	
	2 (7.1)	1–2	*qnrS1* (50.0)	*wt*								Ser-83-Tyr			*wt*	
	1 (3.6)	16	0.0	Ser-83-Leu		Asp-87-His				Ser-80-Ile		Ser-83-Tyr			*wt*	
	1 (3.6)	16	0.0	Ser-83-Leu			Asp-87-Ala			Ser-80-Ile		Ser-83-Tyr			*wt*	
	1 (3.6)	16	0.0	Ser-83-Leu				Asp-87-Tyr		Ser-80-Ile		Ser-83-Tyr			*wt*	
	1 (3.6)	16	0.0	Ser-83-Leu					Asp-87-Gly	Ser-80-Ile		Ser-83-Tyr			*wt*	
	1 (3.6)	32	0.0	Ser-83-Leu	Asp-87-Asn					Ser-80-Ile		Ser-83-Tyr				Ile-464-Phe
	1 (3.6)	32	0.0	Ser-83-Leu	Asp-87-Asn							Ser-83-Tyr	Glu-84-Lys		*wt*	
	1 (3.6)	> 32	0.0	Ser-83-Leu	Asp-87-Asn					Ser-80-Ile		Ser-83-Tyr		Glu-84-Gly	Ser-458-Ala	
Group 2 (*n* = 17)	6 (35.3)	8–16	0.0							Ser-80-Ile		Ser-83-Tyr			*wt*	
	3 (17.6)	16	0.0	Ser-83-Leu	Asp-87-Asn					Ser-80-Ile		Ser-83-Tyr			*wt*	
	3 (17.6)	> 32	0.0	Ser-83-Leu	Asp-87-Asn					Ser-80-Ile		Ser-83-Tyr			Ser-458-Ala	
	3 (17.6)	8	0.0	*wt*								Ser-83-Tyr			*wt*	
	1 (5.9)	32	0.0	Ser-83-Leu		Asp-87-His				Ser-80-Ile		Ser-83-Tyr			*wt*	
	1 (5.9)	32	0.0	*wt*							Ser-80-Arg	Ser-83-Tyr			*wt*	

^a^ PMQR: plasmid-mediated quinolone resistance; QRDR: quinolone resistance determining region; ^b^ Group 1—contained farms that used enrofloxacin; and Group 2—contained farms that did not use enrofloxacin; ^c^ *n* (%), number of isolates (percentage); ^d^ *wt*, wild type without gene mutation; ^e^ Bank means no point mutation.

**Table 6:** Prevalence of PMQR and amino acid changes in the *gyr*A and *par*C genes of *Salmonella* isolates (*n* = 99) from Group 1 and Group 2 and the corresponding MICs of ENR ^a^.

Group ^b^ (*n*)	*n* (%) ^c^	ENR MIC (µg/mL)	PMQR (%)	QRDR Pattern					
				*gyr*A					*par*C
1 (*n* = 50)	28 (56.0)	0.25–1.00	0.00	^d^			Asp-87-Gly		Tyr-57-Ser
	9 (18.0)	0.25–0.50	0.00	Ser-83-Phe					Tyr-57-Ser
	5 (10.0)	0.25–1.00	0.00	*wt* ^e^					Tyr-57-Ser
	4 (8.0)	0.25–0.50	0.00	Ser-83-Phe					*wt*
	2 (4.0)	0.12–0.25	0.00		Ser-83-Tyr				Tyr-57-Ser
	1 (2.0)	0.50	0.00			Ser-83-Ala	Asp-87-Gly		Tyr-57-Ser
	1 (2.0)	0.25	0.00					Asp-87-Asn	Tyr-57-Ser
2 (*n* = 49)	33 (67.3)	0.25–0.50	0.00				Asp-87-Gly		Tyr-57-Ser
	9 (18.4)	0.50–1.00	0.00	Ser-83-Phe					Tyr-57-Ser
	4 (8.2)	0.25	0.00					Asp-87-Asn	Tyr-57-Ser
	1 (2.0)	0.50	0.00	Ser-83-Phe					*wt*
	1 (2.0)	0.25	0.00	*wt*					Tyr-57-Ser
	1 (2.0)	0.25	0.00		Ser-83-Tyr				Tyr-57-Ser

^a^ PMQR: plasmid-mediated quinolone resistance; QRDR: quinolone-resistance determining region; ^b^ Group 1—contained farms that used enrofloxacin; and Group 2—contained farms that did not use enrofloxacin; ^c^ *n* (%), number of isolates (percentage); ^d^ Bank means no point mutation; ^e^ *wt*, wild type without gene mutation.

**Table 7:** Prevalence of PMQR and amino acid changes in the *gyr*A gene of the *Campylobacter* isolates (*n* = 76) from Group 1 and Group 2 and the corresponding MICs of ENR ^a^.

Group ^b^ (*n*)	*n* (%) ^c^	ENR MIC (µg/mL)	PMQR (%)	QRDR Pattern
				*gyrA*
Group 1 (*n* = 50)	50 (100.0)	4–32	0.0	Thr-86-Ile
Group 2 (*n* = 26)	26 (100.0)	4–32	0.0	Thr-86-Ile

^a^ PMQR: plasmid-mediated quinolone resistance; QRDR: quinolone-resistance determining region; ^b^ Group 1—contained farms that used enrofloxacin; and Group 2—contained farms that did not use enrofloxacin; ^c^ *n* (%), number of isolates (percentage).

## Data Availability

Data is contained within the article or Supplementary Material.

## Supplementary Materials

The following supporting information can be downloaded at: https://www.mdpi.com/article/10.3390/foods12112239/s1, Table S1: Sampling of feces and isolation information. Table S2: Specific guideline used for each antimicrobial substance; Table S3: Strains chosen as specific positive control with sequence accession numbers; Table S4: Distribution of the MIC_50_/MIC_90_ values of (fluoro)quinolones among *E. coli* isolates from broiler chickens with or without ENR treatment; Table S5: Distribution of the MIC_50_/MIC_90_ values of (fluoro)quinolones among *Salmonella* isolates from broiler chickens with or without ENR treatment; Table S6: Distribution of the MIC_50_/MIC_90_ values of (fluoro)quinolones among *Campylobacter* isolates from broiler chickens with or without ENR treatment.
