# Supplementary material for: Comparative Studies of Antimicrobial Resistance in Escherichia coli, Salmonella, and Campylobacter Isolates from Broiler Chickens with and without Use of Enrofloxacin
Source: Foods. 2023 Jun 1;12(11):2239. doi: 10.3390/foods12112239 (PMC10252696; doi:10.3390/foods12112239)
Supplement: Supplementary file 1 [file foods-12-02239-s001.zip › Table S1.pdf]

Table S1 Sampling of feces and isolation information

Sampling site	Group	No. of sample /farm	No. of total samples	No. of *E. coli* isolates	No. of *Salmonella* isolates	No. of *Campylobacter* isolates
Broiler farms	Group 1 (n = 7)	n = 55	385	134	40	35
	Group 2 (n = 5)	n = 55	275	173	37	7
Lairage	Group 1 (n = 78)	n = 3–6	319	112	64	49
	Group 2 (n = 60)	n = 3–6	285	8	45	9
Total	-	-	1,364	427	186	100

Group 1: Contained farms that use ENR, and Group 2: Contained farms that do not use ENR.
