# Supplementary material for: Comparative Studies of Antimicrobial Resistance in Escherichia coli, Salmonella, and Campylobacter Isolates from Broiler Chickens with and without Use of Enrofloxacin
Source: Foods. 2023 Jun 1;12(11):2239. doi: 10.3390/foods12112239 (PMC10252696; doi:10.3390/foods12112239)
Supplement: Supplementary file 1 [file foods-12-02239-s001.zip › Table S2.pdf]

**Table S2** Specific guideline used for each antimicrobial substance.

| Bacterial species | Antimicrobial class | Antimicrobials | Breakpoints (μg/mL) | | | Guidelines | |
|---|---|---|---|---|---|---|---|
| | | | S[a] | I | R | Methods | References |
| ***Salmonella*/ *E. coli*** | Combinations | Amoxicillin/clavulanic acid | 8/4 | 16/8 | 32/16 | KRNV4F Sensititre panel | - |
| | Penicillin | Ampicillin | 8 | 16 | 32 | KRNV4F Sensititre panel | - |
| | Cephalosporins | Cephalothin | 8 | 16 | 32 | KRNV4F Sensititre panel | - |
| | | Cefoxitin | 8 | 16 | 32 | KRNV4F Sensititre panel | - |
| | | Ceftiofur | 2 | 4 | 8 | KRNV4F Sensititre panel | - |
| | Amphenicols | Chloramphenicol | 8 | 16 | 32 | KRNV4F Sensititre panel | - |
| | | Florfenicol | -[b] | - | 32 | KRNV4F Sensititre panel | - |
| | Aminoglycosides | Streptomycin | 32 | - | 64 | KRNV4F Sensititre panel | - |
| | | Gentamicin | 4 | 8 | 16 | KRNV4F Sensititre panel | - |
| | | Neomycin | - | - | 16 | KRNV4F Sensititre panel | - |
| | Tetracycline | Tetracycline | 4 | 8 | 16 | KRNV4F Sensititre panel | - |
| | Sulfonamides | Trimethoprim/sulfamethoxazole | 2/38 | - | 4/76 | KRNV4F Sensititre panel | - |
| | Quinolones | Nalidixic acid | 16 | - | 32 | KRNV4F Sensititre panel | - |
| | | Ciprofloxacin | 0.06/1[c] | 0.12–0.5/2 | 1/4 | KRNV4F Sensititre panel | - |
| | | Enrofloxacin | 0.25 | 0.5–1 | 2 | Agar dilution method | [50] |
| | Polymyxin | Colistin | 2 | - | 4 | KRNV4F Sensititre panel | - |
| ***Campylobacter*** | Penicillin | Ampicillin | 8 | 16 | 32 | Agar dilution method | [50] |
| | Tetracycline | Tetracycline | 1/2[d] | - | 2/4 | Campy Sensititre Panel | - |
| | Macrolides | Azithromycin | 0.25/0.5 | - | 0.5/1 | Campy Sensititre Panel | - |
| | | Erythromycin | 4/8 | - | 8/16 | Campy Sensititre Panel | - |
| | Amphenicols | Florfenicol | 4 | - | 8 | Campy Sensititre Panel | - |
| | Aminoglycosides | Gentamicin | 2 | - | 4 | Campy Sensititre Panel | - |
| | Ketolides | Telithromycin | 4 | - | 8 | Campy Sensititre Panel | - |
| | Clinolamides | Clindamycin | 0.5/1 | - | 1/2 | Campy Sensititre Panel | - |
| | Quinolones | Nalidixic acid | 16 | - | 32 | Campy Sensititre Panel | - |
| | | Ciprofloxacin | 0.5 | - | 1 | Campy Sensititre Panel | - |
| | | Enrofloxacin | 0.25 | 0.5–1 | 2 | Agar dilution method | [50] |

[a]S, sensitivity; I, intermediate resistance; R, resistance

[b]-, not supplied in related references

[c]The two values represent *Salmonella* and *E. coli*

[d]The two values represent *Campylobacter jejuni* and *Campylobacter coli*
