# Supplementary material for: Comparative Studies of Antimicrobial Resistance in Escherichia coli, Salmonella, and Campylobacter Isolates from Broiler Chickens with and without Use of Enrofloxacin
Source: Foods. 2023 Jun 1;12(11):2239. doi: 10.3390/foods12112239 (PMC10252696; doi:10.3390/foods12112239)
Supplement: Supplementary file 1 [file foods-12-02239-s001.zip › Table S3.pdf]

Table S3 Strains chosen as specific positive control with sequence accession numbers.

Strain	Species	Resistant gene	GenBank
05k2080	*E. coli*	*qnrA*	OR047762
06K1424	*Citrobacter freundii*	*qnrB*	EU302865
D12-KD-009	*Salmonella*	*qnrD*	OR047765
A16-CF-130-1S-1	*E. coli*	*qnrS*	OR047763
D12-KD-026-S	*Salmonella*	*oqxA*	OR047766
09-C-CF-19	*E. coli*	*aac(6)-Ib-cr*	OR047764
