# Supplementary material for: Comparative Studies of Antimicrobial Resistance in Escherichia coli, Salmonella, and Campylobacter Isolates from Broiler Chickens with and without Use of Enrofloxacin
Source: Foods. 2023 Jun 1;12(11):2239. doi: 10.3390/foods12112239 (PMC10252696; doi:10.3390/foods12112239)
Supplement: Supplementary file 1 [file foods-12-02239-s001.zip › Table S4.pdf]

Table S4. Distribution of MIC50/MIC90 values of (fluoro)quinolones among *E. coli* isolates from broiler chickens with and without ENR treatment.

Group	Source	No. of isolates										
Group 1		Total	Nalidixic acid (μg/mL)									
			16	32	64	128	>128				MIC_{50}	MIC_{90}
	1-day-old	44			2	8	34				>128	>128
	15–25-day-old	105	1	2	2	4	96				>128	>128
	Retail meat	39	7	2	3		27				>128	>128
		Total	Ciprofloxacin (μg/mL)									
			<0.125	0.25	0.5	1	2	4	8	>16	MIC_{50}	MIC_{90}
	1-day-old	44	2	8		1	1	2	17	13	**8**	>16
	15–25-day-old	105	5	4	2	2	6	12	42	32	8	>16
	Retail meat	39	5		2		1	8	10	13	**8**	>16
		Total	ENR (μg/mL)									
			<0.25	0.5	1	2	4	8	16	>32	MIC_{50}	MIC_{90}
	1-day-old	44	7	5	2		1	5	12	12	**16**	>32
	15–25-day-old	105	5	3	4	3	9	26	26	25	8	>32
	Retail meat	39	6		2	1	3	6	9	12	**16**	>32
Group 2		Total	Nalidixic acid (μg/mL)									
			16	32	64	128	>128				MIC_{50}	MIC_{90}
	1-day-old	59			2	8	34				>128	>128
	15–25-day-old	118	1	2	2	4	96				>128	>128
	Retail meat	20	7	2	3		27				>128	>128
		Total	Ciprofloxacin (μg/mL)									
			<0.125	0.25	0.5	1	2	4	8	>16	MIC_{50}	MIC_{90}
	1-day-old	59	2	8		1	1	2	17	13	4	>16
	15–25-day-old	118	5	4	2	2	6	12	42	32	8	>16
	Retail meat	20	5		2		1	8	10	13	4	>16
		Total	ENR (μg/mL)									
			<0.25	0.5	1	2	4	8	16	>32	MIC_{50}	MIC_{90}
	1-day-old	59	7	5	2		1	5	12	12	8	>32
	15–25-day-old	118	5	3	4	3	9	26	26	25	8	>32
	Retail meat	20	6		2	1	3	6	9	12	8	>32

MIC_{50}, MIC of 50% of tested isolates; MIC_{90}, MIC of 90% of tested isolates; Bold MIC parameter indicates the higher MIC_{50}/MIC_{90} value in isolates from Group 1 than Group 1; Bank means no isolate; ENR: enrofloxacin. Group 1: Contained farms that use ENR, and Group 2: Contained farms that do not use ENR.
