# Supplementary material for: Comparative Studies of Antimicrobial Resistance in Escherichia coli, Salmonella, and Campylobacter Isolates from Broiler Chickens with and without Use of Enrofloxacin
Source: Foods. 2023 Jun 1;12(11):2239. doi: 10.3390/foods12112239 (PMC10252696; doi:10.3390/foods12112239)
Supplement: Supplementary file 1 [file foods-12-02239-s001.zip › Table S5.pdf]

Table S5. Distribution of MIC50/MIC90 values of (fluoro)quinolones among *Salmonella* isolates from broiler chickens with and without ENR treatment.

Group	Source	No. of isolates							
Group 1		Total	Nalidixic acid (µg/mL)						
			16	32	64	128	>128	MIC50	MIC90
	1-day-old	25			4	4	17	>128	>128
	15−25-day-old	79	8		6	30	35	128	>128
	Retail meat	30	1		4	13	12	128	>128
		Total	Ciprofloxacin (µg/mL)						
			<0.125	0.25	0.5	1		MIC50	MIC90
	1-day-old	25	13	10	2			<0.125	0.25
	15−25-day-old	79	33	30	15	1		0.25	**0.5**
	Retail meat	30	10	15	4	1		0.25	0.5
		Total	ENR (µg/mL)						
			<0.25	0.5	1	2		MIC50	MIC90
	1-day-old	25	13	6	6			**0.25**	1
	15−25-day-old	79	26	39	12	2		**0.5**	1
	Retail meat	30	8	18	4			0.5	1
Group 2		Total	Nalidixic acid (µg/mL)						
			16	32	64	128	>128	MIC50	MIC90
	1-day-old	26	1		6	6	13	128	>128
	15−25-day-old	56		2	6	15	33	>128	>128
	Retail meat	39	1		3	15	20	>128	>128
		Total	Ciprofloxacin (µg/mL)						
			<0.125	0.25	0.5	1		MIC50	MIC90
	1-day-old	26	21	5				<0.125	0.25
	15−25-day-old	56	25	26	5			0.25	0.25
	Retail meat	39	5	16	18			0.25	0.5
		Total	ENR (µg/mL)						
			<0.25	0.5	1	2		MIC50	MIC90
	1-day-old	26	19	3	4			<0.25	1
	15−25-day-old	56	29	20	7			<0.25	1
	Retail meat	39	5	18	14	2		0.5	1

MIC50, MIC of 50% of tested isolates; MIC90, MIC of 90% of tested isolates; Bold MIC parameter indicates the higher MIC50/MIC90 value in isolates from Group 1 than Group 2; Bank means no isolate; ENR: enrofloxacin. Group 1: Contained farms that use ENR, and Group 2: Contained farms that do not use ENR.
