# Supplementary material for: Comparative Studies of Antimicrobial Resistance in Escherichia coli, Salmonella, and Campylobacter Isolates from Broiler Chickens with and without Use of Enrofloxacin
Source: Foods. 2023 Jun 1;12(11):2239. doi: 10.3390/foods12112239 (PMC10252696; doi:10.3390/foods12112239)
Supplement: Supplementary file 1 [file foods-12-02239-s001.zip › Table S6.pdf]

Table S6. Distribution of MIC_{50}/MIC_{90} values of (fluoro)quinolones among *Campylobacter* isolates from broiler chickens with and without ENR treatment.

Group	Source	No. of isolates										
Group 1		Total	Nalidixic acid (μg/mL)									
			4	32	64	128					MIC_{50}	MIC_{90}
	1-day-old	1	1								1	1
	15–25-day-old	51	11		21	19					64	>64
	Retail meat	48		2	22	24					64	>64
		Total	Ciprofloxacin (μg/mL)									
			<0.125	4	8	16	32				MIC_{50}	MIC_{90}
	1-day-old	1	1								1	1
	15–25-day-old	51	11		34		6				8	32
	Retail meat	48		3	29	16					8	16
		Total	ENR (μg/mL)									
			0.12	0.25–0.5	1	2	4	8	16	32	MIC_{50}	MIC_{90}
	1-day-old	1	1								1	1
	15–25-day-old	51	6		5		20	14	1	5	4	16
	Retail meat	48				3	25	20			4	8
Group 2		Total	Nalidixic acid (μg/mL)									
			16	32	64	128					MIC_{50}	MIC_{90}
	1-day-old	0									NA	NA
	15–25-day-old	16			6	10					>64	>64
	Retail meat	17		2	12	3					64	>64
		Total	Ciprofloxacin (μg/mL)									
			<0.125	4	8	16	32				MIC_{50}	MIC_{90}
	1-day-old	0									NA	NA
	15–25-day-old	16			8	3	5				8	32
	Retail meat	17		3	2	12					**16**	16
		Total	ENR (μg/mL)									
			<0.25	0.5	1	2	4	8	16	>32	MIC_{50}	MIC_{90}
	1-day-old	0									NA	NA
	15–25-day-old	16			10			1		5	4	**32**
	Retail meat	17			4			10	3		**8**	**16**

MIC_{50}, MIC of 50% of tested isolates; MIC_{90}, MIC of 90% of tested isolates; Bold MIC parameter indicates the higher MIC_{50}/MIC_{90} value in isolates from Group 1 than Group 2; Bank means no isolate; ENR: enrofloxacin. Group 1: Contained farms that use ENR, and Group 2: Contained farms that do not use ENR.
